# Synthesis and Evaluation of Paeonol Derivatives as Potential Multifunctional Agents for the Treatment of Alzheimer’s Disease

**DOI:** 10.3390/molecules20011304

**Published:** 2015-01-14

**Authors:** An Zhou, Hongfei Wu, Jian Pan, Xuncui Wang, Jiaming Li, Zeyu Wu, Ailing Hui

**Affiliations:** 1 Institute of Natural Medicine, Hefei University of Technology, Hefei 230009, Anhui, China; E-Mails: anzhou0901@163.com (A.Z.); wu38331451@163.com (Z.W.); 2 Anhui Province Key Laboratory of R&D of Chinese Medicine, Anhui University of Chinese Medicine, Hefei 230038, Anhui, China; E-Mails: wuhongfei2009@126.com (H.W.); wangxuncui@163.com (X.W.); lijiaming2004@sina.com (J.L.)

**Keywords:** Alzheimer’s disease, multifunctional agents, paeonol, acetylcholinesterase inhibitors, metal-chelating, biometal chelators

## Abstract

Alzheimer’s disease (AD) is a progressive neurodegenerative brain disorder characterized by memory loss, language impairment, personality changes and intellectual decline. Taking into account the key pathological features of AD, such as low levels of acetylcholine, beta-amyloid (Aβ) aggregation, oxidative stress and dyshomeostasis of biometals, a new series of paeonol derivatives **5a**–**5d** merging three different functions, *i.e.*, antioxidant, anti-acetylcholinesterase (AChE) activity, metal chelating agents for AD treatment have been synthesized and characterized. Biological assays revealed that compared with paeonol (309.7 μM), **5a**–**5d** had a lower DPPH IC_50_ value (142.8–191.6 μM). **5a**–**5d** could significantly inhibit hydrogen peroxide-induced neuronal PC12 cell death assessed by MTT assay in the concentration range of 5–40 μM. AChE activity was effectively inhibited by **5a**–**5d**, with IC_50_ values in the range of 0.61–7.04 μM. **5a**–**5d** also exhibited good metal-chelating ability. All the above results suggested that paeonol derivatives may be promising multifunctional agents for AD treatment.

## 1. Introduction

Alzheimer’s disease (AD) is a multifaceted neurodegenerative disorder characterized by memory loss, language impairment, personality changes and gradual loss of intellectual ability [1]. Currently, the burden of AD has affected over 35 million people worldwide, increasing with the aging population. If no efficient treatment is discovered, the anticipated number of affected population will increase three-fold by 2025 [2]. Although over 100 years have already elapsed since the discovery of AD, effective treatment methods are still lacking [3]. To date, therapeutic options for symptomatic treatment of AD, which include three acetylcholinesterase (AChE) inhibitors (donepezil, rivastigmine, and galantamine) [4] and an *N*-methyl-d-aspartate receptor (NMDAR) antagonist (memantine) [5], could only result in modest and temporary benefit for memory and cognitive function instead of delaying or stopping the progression of neurodegeneration. Although the molecular etiology of AD is not completely understood, oxidative stress, metal ion deregulation, inflammation, beta-amyloid (Aβ) peptide deposits, τ-protein aggregation, and low levels of acetylcholine (ACh) have been considered to play significant roles in the disease [6,7]. Thus, the development of multitarget agents for simultaneously modulating multiple biological targets involved in AD has clearly emerged as a successful strategy [8,9]. Among the multiple factors inducing AD, a deficiency in ACh as neurotransmitter may play an important role in the regulation of learning and memory processes [10]. ACh is primarily hydrolyzed by AChE, and terminated cholinergic transmission. Furthermore, a broad range of evidences have shown that AChE could accelerate the aggregation of Aβ during the early stages of AD [11,12]. As oxidative stress being also an important player in the pathogenesis and progression of AD, a number of studies has demonstrated that oxidative stress induced by Aβ deposits is associated with the neurodegeneration of the brain [13], and several antioxidants have been tested in clinical trials [14]. Recent studies have indicated that biometals (Cu^2+^, Fe^2+^ and Zn^2+^) also play a very important role in the pathogenesis of AD. High levels of metal ions, like Cu^2+^ and Fe^2+^, have been linked to the production of reactive oxygen species (ROS), which promotes oxidative stress thus contributing to AD pathogenesis [15]. Thus, the modulation of these biometals in the brain is also a viable alternative to treat AD patients. In view of multi-pathogenesis of AD, more efforts are directed to develop multi-target-directed ligands (MTDLs), which has been proven to be a successful strategy for the design of new drugs for AD [16,17]. Paeonol (2-hydroxy-4-methoxy-acetophenone), a natural phenolic component isolated from the root bark of Moutan Cortex (*Paeonia suffruticosa*), possesses well known antioxidant and anti-inflammatory activities. Recent evidence has suggested that paeonol could attenuate neurotoxicity and ameliorate cognitive impairment [18]. It is also proved that paeonol ameliorates neuronal damage via the inhibition of microglia-mediated inflammation and oxidative stress-induced neuronal damage. In a rat model of AD, treatment with paeonol can protect against many alterations resulting from administration of Aβ_1–42_ [19,20]. Our previous research also showed that paeonol could protect against glutamate-induced PC12 cell death via a ROS mitochondrial pathway [21]. Considering the cholinergic hypothesis, the importance of biometal ions and oxidative stress in the pathological process of AD [22], we are interested in the development of novel paeonol derivatives as potential multifunctional agents for the treatment of AD. In the present study, a series of paeonol derivatives were synthesized and then their antioxidant activities, acetylcholinesterase inhibitory, metal chelating effects and protective effects on H_2_O_2_-induced PC12 cell death were investigated.

## 2. Results and Discussion

### 2.1. Chemical Synthesis

**Scheme 1:**
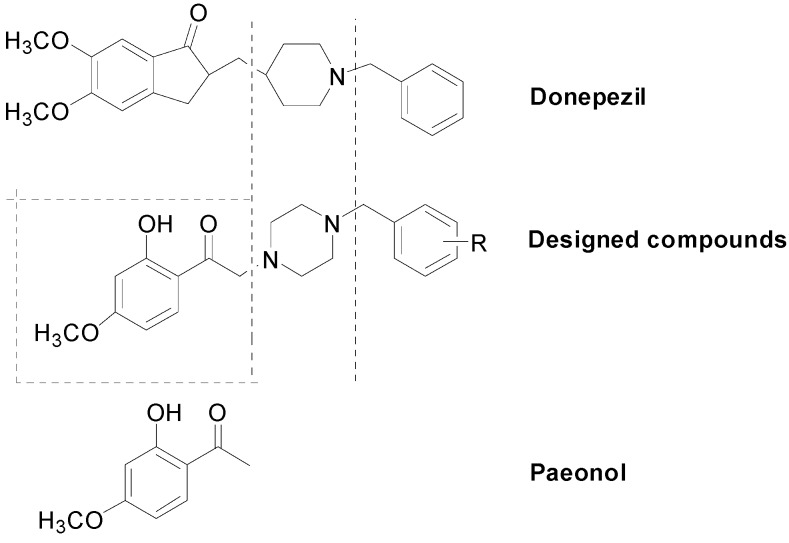
Design of new donepezil-like analogs based on the structure of paeonol and donepezil.

**Scheme 2:**
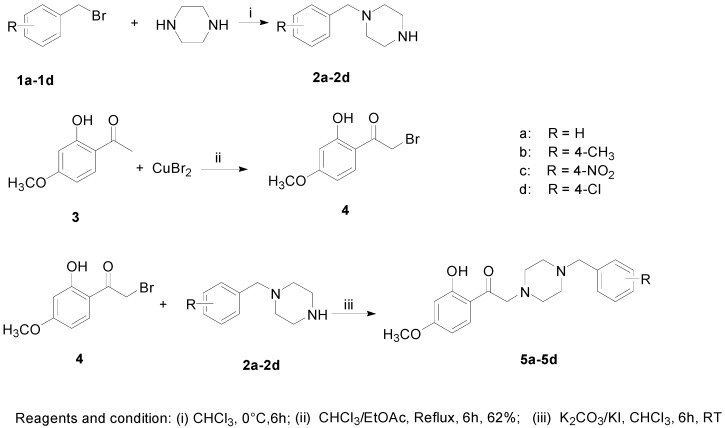
Synthetic route of the title compounds.

Donepezil is the first-line drug to prevent AD. Based on the structure of paeonol and donepezil, a series of new donepezil-like analogs were designed as depicted in Scheme 1. The synthetic pathway of new paeonol derivatives was shown in Scheme 2. Firstly, various monosubstituted N-alkyl piperazines (**2a**–**2d**) and α-brominated paeonol (**4**) were prepared according to the slightly modified literatures [23,24], respectively. Once the intermediate was available, *N*-alkyl piperazines (**2a**–**2d**) reacted with **4** in the presence of K_2_CO_3_/KI to yield the desired **5a**–**5d**.

### 2.2. Biological Evaluation

#### 2.2.1. Free Radical Scavenging Activity *in Vitro* (DPPH Assay)

Paeonol is well known for its antioxidant property [25]. Based on their interaction with DPPH (2,2-diphenyl-1-picrylhydrazyl) free radical, antioxidant activities of the new paeonol derivatives were screened. Concentrations caused 50% loss of the initial DPPH free radical scavenging (IC_50_) for paeonol derivatives **5a**–**5d**, which are listed in Table 1. Paeonol is used as the positive control. It could be observed that all the paeonol derivatives showed greater DPPH radical scavenging activity compared with paeonol. Their IC_50_ values were in the range of 142.79–191.64 μM. In the same condition, paeonol has a value of 309.67 μM. The above results indicated that paeonol derivatives **5a**–**5d** have good radical scavenging and antioxidant properties. Among the four derivatives, the IC_50_ values of **5a** and **5b** are slightly lower than those of **5c** and **5d**.

**Table 1:** Antioxidant and anticholinesterase activities of the Paeonol derivatives **5a**–**5b**.

Compounds	Molecular Weight	DPPH Scavenging (^a^ IC_50_, μM)	AChE Inhibition (^b^ IC_50_, μM)
**5a**	340.1	142.79 ± 8.83	1.59 ± 0.011
**5b**	354.2	157.85 ± 6.88	0.61 ± 0.009
**5c**	385.4	191.64 ± 8.06	7.04 ± 0.032
**5d**	374.1	187.32 ± 13.73	2.63 ± 0.153
**Paeonol**	166.2	309.67 ± 5.41	nt
**Donepezil**	379.4	nt	0.053 ± 0.003

Notes: ^a^: Concentrations caused 50% loss of the initial DPPH free radical scavenging; data are expressed as the mean ± SD, *n* = 3; ^b^: AChE from electric eel, *n* = 3; nt: Not tested.

#### 2.2.2. Inhibitory Effect on PC12 Cell Death Induced by Oxidative Stress

**Figure 1:**
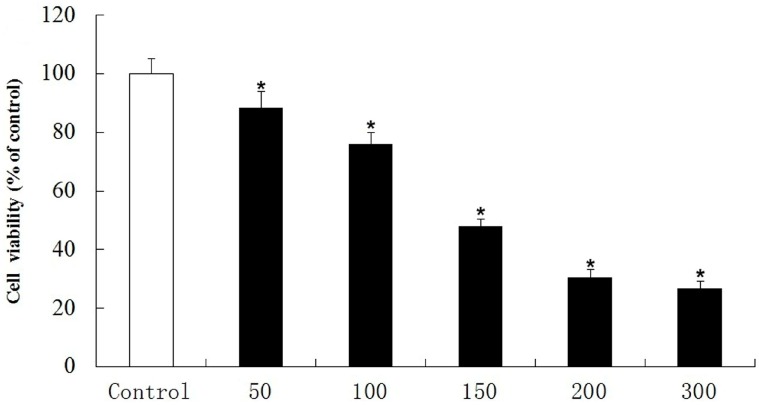
The MTT assay was used to detect cytotoxicity for various concentrations of H_2_O_2_. Data are shown as mean ± SD (*n* = 10). (*) compared with control group (*p* < 0.05 one-way ANOVA).

**Figure 2:**
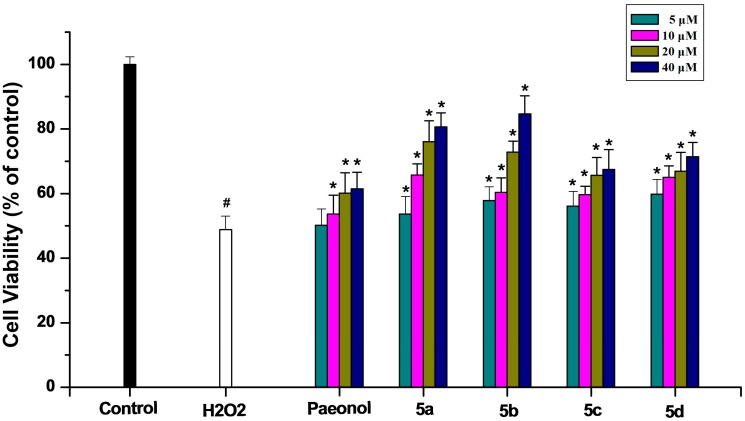
Cytoprotective effects of paeonol and target compounds **5a**–**5d** in PC12 cells under H_2_O_2_-induced oxidative stress. PC12 cells exposed to 150 µM H_2_O_2_ for 5 h after being pretreated with test compounds for 3 h. Cell viability was determined using the MTT method. The viability of control cells untreated with test compounds and H_2_O_2_ was defined as 100%. Data are shown as mean ± SD (*n* = 10). (*) compared with H_2_O_2_-treated group (*p* < 0.05); (#) compared with control group (*p* < 0.05 two-way ANOVA).

The MTT assay was used to identify the degree of cell death after treatment with H_2_O_2_ at various concentrations. From Figure 1, it could be seen that the PC12 cell viability decreased in a concentration dependent manner. Compared with control group, the cell viability decreased to 47.1% after being exposed to 150 μM H_2_O_2_ for 5 h and then 150 μM H_2_O_2_ was chosen in the following experiments. The cell viability under the treatment of different concentrations of paeonol and its derivatives are listed in Figure 2. Paeonol treatment did not cause any apparent effects on PC12 cells when its concentration was 5 μM. However, **5a**–**5d** showed stronger protection effects at the same concentrations, which is consistent with the results of DPPH assay. Even if the concentration of paeonol was increased to 40 μM, its protective effect was only 58.9%. Meanwhile, **5a**–**5d** gave 80.7%, 84.2%, 61.8%, and 64.1% reductions, respectively. The above results demonstrated that compounds **5a** and **5b** showed more potent activity at higher concentrations.

**Figure 3:**
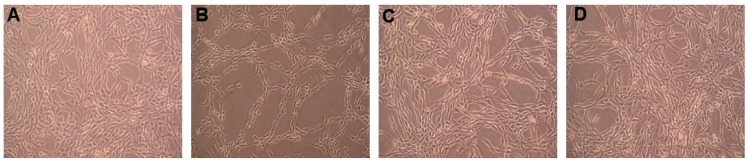
Identification of morphological cell changes with phase-contrast microscopy. (**A**) PC12 control cells; (**B**) PC12 cells exposed to 150 μM H_2_O_2_ for 5 h. There is a significant decrease in the cell number; (**C**,**D**) PC12 cells preincubated with (C) paeonol (40 µM) or (D) **5b** (40 μM) for 3 h and then exposed to 150 μM H_2_O_2_ for 5 h. All the photos were taken 5 h after exposure to H_2_O_2_ (visualized under a light microscope at ×100 magnification).

In comparison to the untreated group (Figure 3A), PC12 cells exhibited a marked decrease in cell number and morphological alterations such as cell shrinkage and membrane blebbing after being treated with 150 μM H_2_O_2_ for 5 h (Figure 3B). Compared with the H_2_O_2_ treated group (Figure 3B), PC12 cells pretreated with 40 µM paeonol (Figure 3C) and 40 µM **5b** (Figure 3D) maintained normal cell morphology, because of the mitigation of such morphological features. Meanwhile, the results of cell viability also clearly indicated that **5a**–**5d** could attenuate the cytotoxic effects on PC12 cells induced by H_2_O_2_ (Figure 2).

#### 2.2.3. Anticholinesterase Activity

All the compounds **5a**–**5d** were evaluated against AChE (from electric eel) using the method of Ellman *et al.* [26], with donepezil as the reference compound. As the IC_50_ values shown in Table 1, all the compounds demonstrated moderate inhibition activities with IC_50_ values in the range of micromolar to sub-micromolar. Among the tested derivatives, **5b** with para methyl substituent on phenyl ring was the most potent one in this series (IC_50_ = 0.61 μM). The other three compounds presented poor inhibitory activity, which had no substituent (**5a**) or electron-withdrawing groups (**5c** and **5d**) on phenyl ring.

#### 2.2.4. Molecular Docking

**Figure 4:**
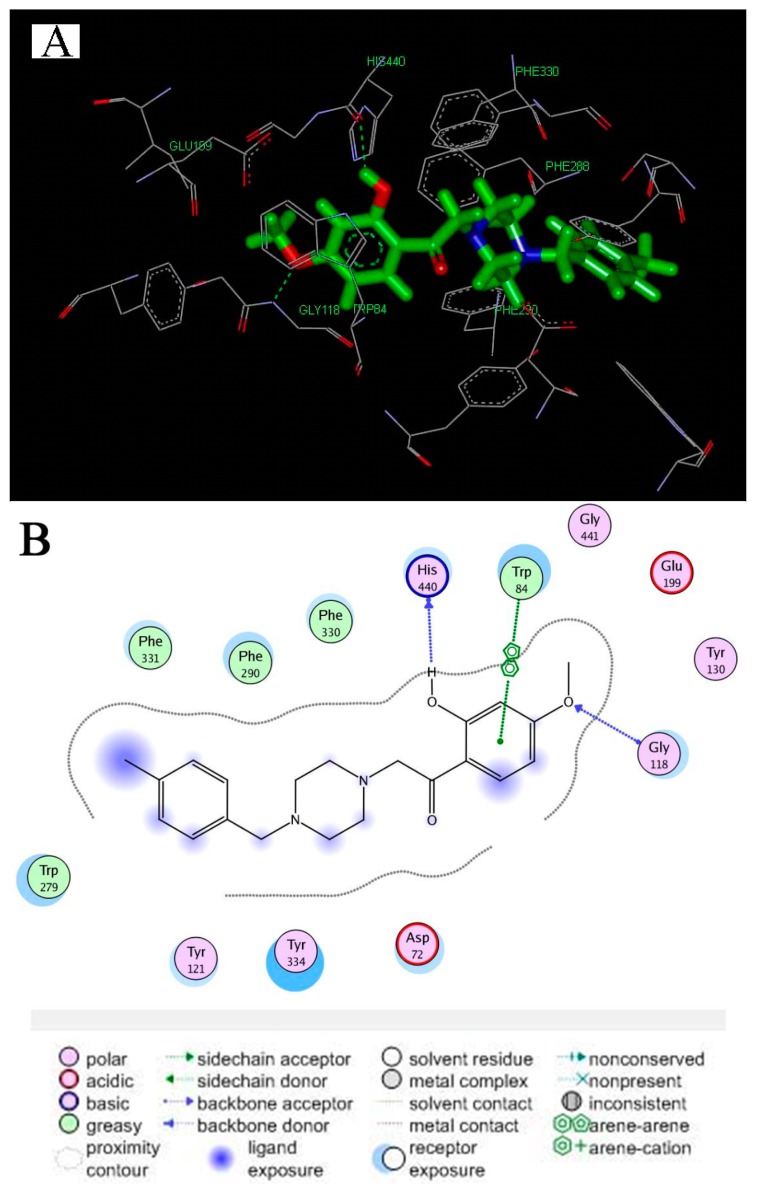
Representations of the molecular model of the complex formed between compound 5b and TcAChE (PDB code: 1EVE). (**A**) 3D representation of the ligand-enzyme binding interactions (hydrogen bonds as green dashed lines); (**B**) 2D schematic representation of the hydrogen bonding and hydrophobic interactions.

To explore a possible interacting mode of the compound **5b** with TcAChE (PDB code: 1EVE), a molecular modeling study was performed with the MOE software program. As shown in Figure 4A,B, compound **5b** moiety occupied the peripheral anionic site (PAS) of enzyme and possessed potential hydrophobic interaction with residues Tyr334, Phe331, Phe331, Phe290 and Tyr279. Moreover, the linker was long enough for the paeonol substituted group arriving in the catalytic active site (CAS) of AChE, it adopted an appropriate orientation for its binding to CAS via a π-π interaction with Trp84 and potentially induced a hydrogen bond interaction with residues His440 and Gly118. The simultaneous binding of **5b** with the CAS and PAS of TcAChE provided an explanation for its demonstrated moderate inhibitory activity for AChE and thus revealed a mixed-type inhibition for this compound.

#### 2.2.5. Metal-Chelating Study

**Figure 5:**
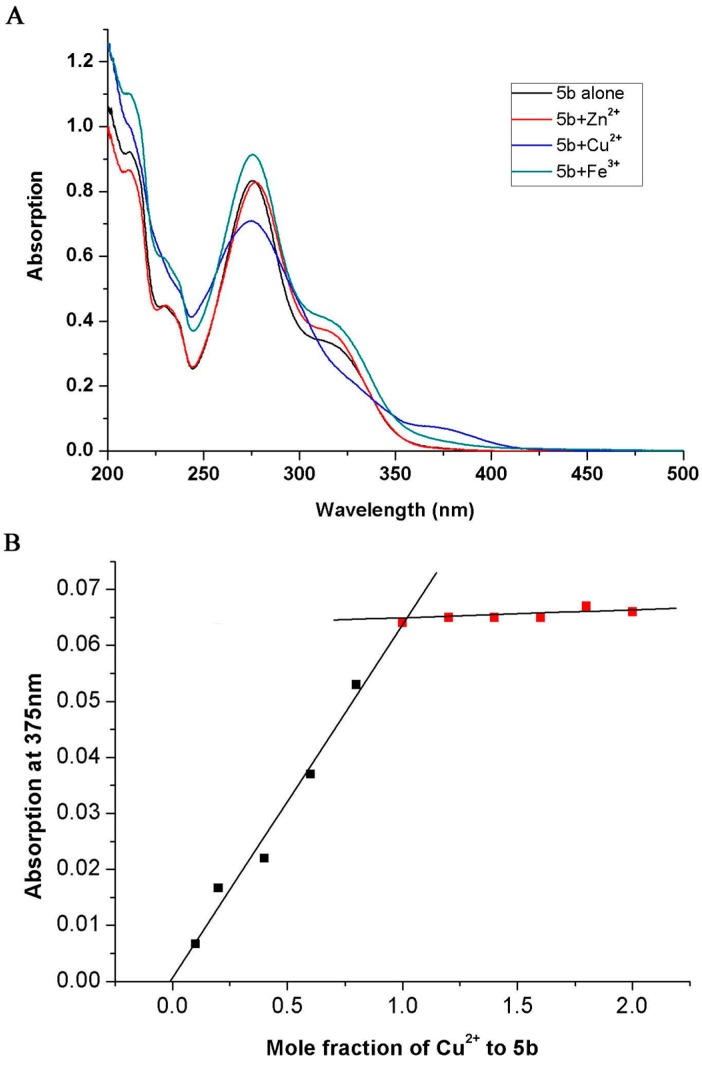
(**A**) The UV spectrum of compound **5b** (40 μM) alone or in the presence of FeSO_4_, CuCl_2_ or ZnCl_2_ (40 μM for all the metals in methanol); (**B**) Determination of the stoichiometry of complex **5b**-Cu (II) by using molar ratio method through titrating methanol solution of compound **5b** with ascending amounts of CuCl_2_. The final concentration of test compound **5b** was 40 μM, and the final concentration of Cu^2+^ ranged from 2–80 μM.

The chelating ability of the most potent compound **5b** for the biologically relevant metal ions such as Cu^2+^, Fe^2+^ and Zn^2+^ in methanol was studied by UV-vis spectrophotometer [27,28]. Figure 5A showed that UV absorption of compound **5b** in methanol changed with addition of Cu^2+^ or Fe^2+^, while little change occurring after titrating with Zn^2+^. The electronic spectra of **5b** exhibited a blue shift (the peak at 275 nm shifted to 271 nm) after addition of CuCl_2_ and the absorbance dropped dramatically, indicating that **5b** could interact with Cu^2+^ effectively. The absorption intensity change could also be observed after adding FeSO_4_. The stoichiometry of complex **5b**-Cu (II) was determined using the molar ratio method [29,30], by titrating solutions of compound **5b** with ascending amounts of CuCl_2_. The UV spectra were used to obtain the absorbance of **5b** complex and different concentrations of CuCl_2_ at 375 nm. As shown in Figure 5B, absorbance firstly increased and then reached the saturation point. The point for the two straight lines intersected at the mole fraction of 0.98, indicating a 1:1 stoichiometry for the **5b**-Cu(II) complex.

## 3. Experimental Section

### 3.1. Chemistry

Paeonol (purity > 99%) was purchased from Nanjing Ze-Lang Medical Technology Co., Ltd (Nanjing, China). In the synthesis experiments, all the reagents and solvents were reagent grade and used without further purification unless otherwise indicated. All the reactions were monitored using TLC. Column chromatography was performed using silica gel (200–300 mesh). All yields of the products refer to isolated yield. IR spectra were obtained on Thermo Scientific Nicolet 8700 system. NMR spectra were obtained on Agilent VNMRS (600 MHz) with TMS as internal standard, and the chemical shifts were recorded in ppm along with coupling constants (*J* values) in Hz. Multiplicities were designated as singlet (s), broad singlet (br), doublet (d), triplet (t) and multiplet (m). Mass spectra were recorded by electrospray ionization (ESI) on a Thermo LCQ Advantage MAX spectrometry with direct insertion probe. Elemental analysis (EA) was obtained by a PE 2400 (Perkin Elmer, Waltham, MA, USA) analyzer.

### 3.2. Synthesis

General procedure for the synthesis of **2a**–**2d**: Anhydrous piperazine (18.0 g, 0.21 mol) was dissolved in chloroform (60 mL). Benzyl bromide **1a**–**1d** (0.05 mol) in chloroform (30 mL) was added dropwise to the solution at 0 °C. The reaction mixture was stirred for about 6 h at room temperature. Then, the mixture was washed with 5% K_2_CO_3_ solution (80 mL × 2) and water (80 mL × 4). The organic layer was dried over anhydrous Na_2_SO_4_. The filtrate was evaporated *in vacuo* and the residue was recrystallized from *n*-hexane to give the desired products **2a**–**2d**.

*1-Benzylpiperazine* (**2a**): Yield 77%, ^1^H-NMR (CDCl_3_, 600 MHz): δ 7.28–7.31 (m, 4H), 7.22–7.24 (m, 1H), 3.47 (s, 2H), 2.85–2.89 (m, 4H), 2.38–2.43 (m, 4H), 1.82 (br, 1H) ppm.

*1-(4-Methylbenzyl)piperazine* (**2b**): Yield 77%, ^1^H-NMR (CDCl_3_, 600 MHz): δ 7.16–7.22 (m, 2H), 7.06–7.11 (m, 2H), 3.45 (s, 2H), 2.82–2.87 (m, 4H), 2.37–2.42 (m, 4H), 2.31 (s, 3H), 1.74 (br, 1H) ppm.

*1-(4-Nitrobenzyl) piperazine* (**2c**): Yield 22%,^1^H-NMR (CDCl_3_, 600 MHz): δ 8.21 (d, *J* = 8.9 Hz, 2H), 7.60 (d, *J* = 9.0 Hz, 2H), 3.58 (s, 2H), 3.02–3.15 (m, 4H), 2.58–2.74 (m, 4H) ppm.

*1-(4-Chlorobenzyl) piperazine* (**2d**): Yield 53%, ^1^H-NMR (CDCl_3_, 600 MHz): δ 7.25–7.29 (m, 4H), 3.45 (s, 2H), 2.89–2.94 (m, 4H), 2.39–2.45 (m, 4H), 1.73 (br, 1H) ppm.

Synthesis of 2-bromo-1-(2-hydroxy-4-methoxy-phenyl)-ethanone **4**: Powdered cupric bromide (13.4 g, 60 mmol) was added into a solution of paeonol (5.0 g, 30 mmol) in chloroform (100 mL) and ethyl acetate (100 mL). The mixture was refluxed at 90 °C for 6 h until the reation was complete (monitored by TLC). After cooling, the mixture was filtered. The filtrate was washed with H_2_O (150 mL × 4), dried over anhydrous Na_2_SO_4_. The filtrate was evaporated *in vacuo* and the residue was purified by chromatography on silica gel column with ethyl acetate/petroleum ether as the elution. The resulting bromidated panonol **4** (4.5 g) was obtained as white powder. Yield 62%, ^1^H-NMR (CDCl_3_, 600 MHz): δ 6.75–8.15 (s, 3H), 3.95 (s, 3H), 2.59 (s, 2H) ppm.

General procedure for the synthesis of **5a**–**5d**: 2-Hydroxy-4-methoxy-1-(2-bromo)-acetophenon **4** (2.1 g, 5 mmol) and *N*-substituted benzyl piperazine **2a**–**2d** (10 mmol) were dissolved in chloroform (60 mL). Anhydrous K_2_CO_3_ (3.46 g, 25 mmol) and catalytic KI (0.17 g, 0.5 mmol) were added to the solution. The reaction mixture was stirred for about 6 h at room temperature. Then, the solution was washed with H_2_O (60 mL × 3). The organic layer was dried, filtered and evaporated. The resulting crude product was purified by using flash column chromatography on silica gel with ethyl acetate/petroleum ether as elution solvent to give yellow oil **5a**–**5d**.

*2-(4-Benzylpiperazin-1-yl)-1-(2-hydroxy-4-methoxyphenyl) ethanone* (**5a**): Yield 78%, yellow oily product. IR (film): ν 3026.0, 2938.1, 2813.0, 1715.2, 1616.1, 1507.7, 1441.8, 838.9, 747.0 cm^−1^. ^1^H-NMR (CDCl_3_, 600 MHz): δ 9.96 (s, 1H), 7.79 (d, *J* = 6.0 Hz, 1H), 7.28 (d, *J* = 6.0 Hz, 4H), 7.22 (d, *J* = 6.0 Hz, 1H), 6.40 (d, *J* = 6.0 Hz, 1H), 6.38 (t, *J* = 6.0 Hz, 2H), 3.77 (s, 3H), 3.62 (s, 2H), 3.49 (s, 2H), 2.62(m, 4H), 2.52 (m, 4H) ppm. ^13^C-NMR (CDCl_3_, 150 MHz): δ 168.71, 140.35, 134.49, 131.83, 130.90, 129.81, 110.32, 104.00, 65.48, 58.46, 55.73, 55.15 ppm. ESI-MS: *m*/*z* 341.2 [M+H]^+^. Anal. Calcd. for C_20_H_24_N_2_O_3_: C, 70.56; H, 7.11; N 8.23; O 14.10. Found: C, 70.32; H, 7.18; N, 8.36.

*1-(2-Hydroxy-4-methoxyphenyl)-2-(4-(4-methylbenzyl) piperazin-1-yl) ethanone* (**5b**): Yield 57%, pale yellow solid. IR (KBr): ν 2939.0, 2812.8, 1701.3, 1611.9, 1506.7, 1441.1, 829.9 cm^−1^. ^1^H-NMR (CDCl_3_, 600 MHz): δ 9.96 (s, 1H), 7.84 (d, *J* = 12.0 Hz, 1H), 7.20 (d, *J* = 12.0 Hz, 2H), 7.13 (d, *J* = 12.0 Hz, 2H), 6.44 (d, *J* = 6.0 Hz,1H), 6.41 (t, *J* = 6.0 Hz, 1H), 3.83 (s, 3H), 3.66 (s, 2H), 3.49 (s, 2H), 2.65 (m, 4H), 2.51 (m, 4H), 2.33 (s, 3H) ppm. ^13^C-NMR (CDCl_3_, 150 MHz): δ 169.79, 164.30, 161.29, 132.00, 129.90, 127.11, 124.19, 120.87, 106.51, 102.98, 96.60, 95.44, 91.13, 58.65, 57.85, 50.76, 48.36, 47.72, 16.37 ppm. ESI-MS: *m*/*z* 355.3 [M+H]^+^. Anal. Calcd. for C_21_H_26_N_2_O_3_: C, 71.16; H, 7.39; N, 7.90. Found: C, 70.24; H, 7.40; N, 7.98.

*1-(2-Hydroxy-4-methoxyphenyl)-2-(4-(4-nitrobenzyl) piperazin-1-yl) ethanone* (**5c**): Yield 31%, yellow solid. IR (KBr): ν 2942.1, 2809.6, 1632.6, 1615.4, 1515.6, 1350.8, 858.4, 829.5 cm^−1^. ^1^H-NMR (CDCl_3_, 600 MHz): δ 8.25 (d, *J* = 12.0 Hz, 2H), 7.88 (d, *J* = 12.0 Hz, 1H), 7.58 (d, *J* = 6.0 Hz, 2H), 6.51 (t, *J* = 6.0 Hz, 1H), 6.47 (s, 1H), 3.90 (s, 3H), 3.77 (s, 2H), 3.68 (s, 2H), 2.74 (m, 4H), 2.64 (m, 4H) ppm. ^13^C-NMR (CDCl_3_, 150 MHz): δ 161.36, 160.30, 142.40, 141.22, 126.87, 124.75, 118.78, 103.01, 96.46, 57.17, 50.80, 48.37, 47.94 ppm. ESI-MS: *m*/*z* 444.10 [M+H]^+^. Anal. Calcd. for C_20_H_23_N_3_O_5_: C, 62.33; H, 6.01; N, 10.90. Found: C, 62.41; H, 5.98; N, 10.97.

*2-(4-(4-Chlorobenzyl) piperazin-1-yl)-1-(2-hydroxy-4-methoxypheny) ethanone* (**5d**): Yield 44%, pale yellow solid. IR (KBr): ν 2936.6, 2811.8, 1637.9.1, 1510.5, 1489.6, 1455.1, 815.3 cm^−1^. ^1^H-NMR (CDCl_3_, 600 MHz): δ 7.82 (d, *J* = 12.0 Hz, 1H), 7.23–7.26 (m, 4H), 6.42 (t, *J* = 6.0 Hz, 1H), 6.40 (t, *J* = 6.0 Hz, 1H), 3.82 (s, 3H), 3.66 (s, 2H), 3.48 (s, 2H), 2.64 (m, 4H), 2.53 (m, 4H) ppm. ^13^C-NMR (CDCl_3_, 150 MHz): δ 168.75, 138.98, 135.53, 134.43, 133.05, 131.06, 110.37, 103.96, 64.66, 58.16, 55.74, 55.15 ppm. ESI-MS: *m*/*z* 375.2 [M+H]^+^. Anal. Calcd. for C_20_H_23_ClN_2_O_3_: C, 64.08; H, 6.18; N, 7.47. Found: C, 64.14; H, 6.02; N, 7.42.

### 3.3. DPPH Radical Scavenging Activity

The free radical scavenging capacities of paeonol and target compounds **5a**–**5d** were evaluated by the recognized method. The stable radical DPPH was used as a reagent in this spectrophotometric assay. Two milliliters of various concentrations of the test compounds (0.08–0.8 mM) in methanol were added into 2 mL of 0.3 mM DPPH methanol solution; therefore, the appropriate amount of methanolic solution of each the test compounds was added to obtain cuvette concentrations ranging from 0.04–0.4 mM. After a 60 min incubation period at room temperature in the dark, the absorbance was read at 517 nm against the corresponding blank. Inhibition of free radical DPPH in percent (*I*%) was calculated using the following Equation (1):

(1) $$I\%=(\frac{A_{\text{blank}}-A_{\text{sample}}}{A_{\text{blank}}})\times 100$$

Where A_blank_ is the absorbance of DPPH solution (control solution) against the blank, and A_sample_ is the absorbance of the tested compound against the blank. Concentrations caused 50% loss of the initial DPPH free radical scavenging.

### 3.4. Protection of PC12 Cells against Hydrogen Peroxide-Induced Damage

#### 3.4.1. Cell Culture

PC12 (rat pheochromocytoma) cells were obtained from the Chinese Academia Sinica (Shanghai, China). Cells were cultured at 37 °C under a humidified 5% CO_2_ atmosphere in RPMI1640 medium supplemented with 10% (*v*/*v*) fetal bovine serum, 100 U/mL penicillin G and 100 μg/mL streptomycin. Before treatment, the differentiated PC12 cells were plated at appropriate density on 96-well culture plates and cultured for 24 h. In all experiments, cells were preincubated with different concentrations of paeonol and its derivatives dissolved in DMSO and diluted with DMEM with final concentration of DMSO less than 0.1% for 3 h, and later, H_2_O_2_ was added to the medium. Not all of the compounds were removed after the addition of H_2_O_2_. The sample was prepared as stock solution in DMSO and diluted with DMEM with the final concentration of DMSO less than 0.1%, 0.1% (*v*/*v*) DMSO having no protective or toxic effect by itself.

#### 3.4.2. Cell Viability Assay

Cell viability following exposure to H_2_O_2_ was measured by the MTT reduction assay according to our previous study [21]. Before the treatment, differentiated PC12 cells were seeded on 96-well culture plates at a density of 1 × 10^5^ cells/well and cultured for 24 h. Then, the medium was changed and cells were treated with paeonol and its derivatives (5 µM, 10 µM, 20 µM, 40 µM) for 3 h, respectively. After this time, the cells were exposed to 150 µM H_2_O_2_ for 5 h. Then, 20 µL of MTT solution (5 mg/mL) was added to 200 µL culture medium of each well. Four hours later, the medium was removed, and the resultant formazan product was dissolved by addition of 150 µL of DMSO. The absorbance of MTT was measured at 570 nm with a microplate reader (ELX 800; Bio-TEK instruments, Inc., Winooski, VT, USA). The results were expressed as percentage of cell viability (%), assuming that of control cells is 100%.

### 3.5. In Vitro AChE Inhibition Assay

Acetylcholinesterase (AChE, E.C.3.1.1.7, from the electric eel), 5,5'-dithiobis-(2-nitrobenzoic acid) (DTNB), acetylthiocholine iodide (ATCI) and donepezil hydrochloride were purchased from Sigma Aldrich. The inhibitory activities against AChE of paeonol and target compounds **5a**–**5d** were performed according to the method developed by Ellman *et al.* [26], using donepezil as the reference compound. All the *in vitro* assays were carried out in 0.1 M KH_2_PO_4_/K_2_HPO_4_ buffer (pH 8.0) using a Shimadzu UV-2550 UV-Vis Spectrophotometer. Paeonol and **5a**–**5d** were dissolved in DMSO and then diluted by phosphate buffer (pH 8.0) to seven different concentrations in the range of 10^−3^–10^−9^ M. AChE solutions were prepared in double-distilled water to 2.0 units/mL in 2 mL aliquots. The assay medium contained phosphate buffer, pH 8.0 (1 mL), 50 µL of 0.01 M DTNB, 10 µL of enzyme and 50 µL of acetylthiocholine iodide. Test compounds were added to assay solution and preincubated at 37 °C with enzyme for 15 min, followed by the addition of substrate. Activity was determined by measuring the increase in absorbance at 412 nm at 1 min intervals at 37 ± 0.2 °C. For determining the blank value, 50 µL buffer replaced the AChE solution. Each concentration was assayed three times and 50% inhibitory concentration (IC_50_) was calculated graphically from inhibition curves.

### 3.6. Molecular Docking

To further investigate the interaction mode for AChE, molecular docking study was carried out by Molecular Operating Environment (MOE 2008.10) software package. The crystal complex (PDB code: 1EVE) was downloaded from the protein data bank [31]. The protein was added to hydrogens and calculated with partial charges (Gasteiger methodology). Energy minimization (MMFF94x, gradient: 0.05) was performed. The structure was energy minimized in AMBER force filed to an RMSD of 0.05 Å where the implicit solvated environment was specified and the stabilized conformation was saved. The 3D structure of the strongest AChE inhibitor **5b** was built using the builder interface of MOE program, and docked into the active site of the protein after energy minimized. All parameters were left as default values. Finally, the conformation with the lowest docking score was selected for analyzing the interactions between the AChE and **5d**.

### 3.7. Metal-Chelating Studies

The metal-chelating studies were carried out in methanol using a UV-vis spectrophotometer (SHIMADZC UV-2550PC). FeSO_4_, CuCl_2_ or ZnCl_2_ solution was prepared to 200 μM using methanol. The test compounds **5a**–**5d** were also dissolved in methanol to 400 μM. To investigate the metal binding ability of compounds, a mixture of 100 μL test compound solution was added with 200 μL FeSO_4_ solution (CuCl_2_ or ZnCl_2_) and 700 μL methanol in a 1 cm quartz cuvette. The solution was incubated for 30 min at room temperature and then the absorption spectra were recorded with wavelength ranging from 200–500 nm. The stoichiometry of complex **5b**-Cu (II) was determined using the molar ratio method [29,30], by titrating solutions of compound **5b** with ascending amounts of CuCl_2_. The final concentration of tested compound **5b** was 40 μM, and the final concentration of Cu^2+^ ranged from 4–80 μM. The recorded UV spectra data were treated by numerical subtraction of CuCl_2_ and **5b** at corresponding concentrations, and then plotted *versus* the mole fraction of **5b**.

### 3.8. Statistical Analysis

Data were represented as mean ± SD. Comparisons between several groups were performed by analysis of variance (ANOVA). A value of *p* < 0.05 was considered to be statistically significant.

## 4. Conclusions

In summary, a series of novel paeonol derivatives **5a**–**5d** were designed, synthesized and characterized as multifunctional agents for the treatment of AD. Their structures were identified by means of spectral data and elemental analysis. Their antioxidant activities were evaluated by DPPH radical-scavenging activity assays and their protective effects on H_2_O_2_-induced cytotoxicity in PC12 cells by the MTT assay method. Compared with paeonol, **5a**–**5d** possessed stronger antioxidant activity and protective effects on H_2_O_2_-induced PC12 cell death. In addition, these compounds also had moderate AChE inhibition properties, with IC_50_ values ranging from micromolar to sub-micromolar. Among them, compound **5b**, with para methyl substituent on phenyl ring was found to bind to Cu^2+^ and assemble into a metal complex. Further investigations of AD candidates based on these results are in progress.
